# Lower Limb Metaphyseal Bone Is Lost in Men with Coeliac Disease and Does Not Relate to Parathyroid Status

**DOI:** 10.1155/2016/4131794

**Published:** 2016-09-08

**Authors:** Michael W. J. Davie, Sally F. Evans, Christopher A. Sharp

**Affiliations:** ^1^Charles Salt Centre for Human Metabolism, Robert Jones and Agnes Hunt Orthopaedic Hospital, Oswestry SY10 7AG, UK; ^2^Institute of Medicine, University of Chester, Bache Hall, Chester CH1 4BJ, UK; ^3^Institute of Medicine, University Centre Shrewsbury, University of Chester, The Guildhall, Frankwell Quay, Shrewsbury, Shropshire SY3 8HQ, UK

## Abstract

*Aims*. To investigate regional lower limb bone density and associations with weight, PTH, and bone breakdown in coeliac men.* Methods*. From whole body DXA scans bone mineral density (BMD) was measured in 28 coeliac men, in the lower limb (subdivided into 6 regions, 3 being metaphyseal (mainly trabecular) and 2 diaphyseal (mainly cortical)). BMD at femoral neck (FN) and lumbar spine L2–4, body weight, height, serum calcium, alkaline phosphatase, parathyroid hormone (PTH), and urinary calcium and NTx/Cr, a measure of bone breakdown, were also measured. Age matched healthy men provided values for BMD calculation of *z* and *T* scores and for biochemical measurements.* Results*. Low BMD *z* scores were found at metaphyseal regions in the leg (*p* < 0.001) and in the FN (*p* < 0.05). The distal metaphyseal region BMD in the leg was lower than spine or FN (*p* < 0.05). PTH, urinary calcium/creatinine, and urinary NTx/Cr were similar to controls. Both metaphyseal and diaphyseal BMD *z* scores were associated with body weight (*p* < 0.02), but not with either PTH or urinary NTx/Cr.* Conclusions*. Low BMD lower limb regions comprising mostly trabecular bone occur early in CD and in the absence of elevated PTH or increased bone resorption. Low BMD is associated with low body weight.

## 1. Introduction

Bone deficit in coeliac disease (CD) is well recognised [1]. This has been ascribed to the secondary hyperparathyroidism consequent upon the malabsorption of calcium and of vitamin D [2]. At sites such as the distal forearm areal low bone mineral density (BMD) *z* scores were related to higher parathyroid hormone (PTH) values although no correlation with 25-hydroxyvitamin D (25(OH)D) was found [2]. Classical studies showing increased bone turnover markers in coeliac disease were obtained before the advent of serological testing made the diagnosis of CD less invasive [3, 4] and may have detected patients at a relatively advanced stage. More recent reports find that hyperparathyroidism is present in fewer than 30% of CD patients at diagnosis [5–8]. Low BMD, an important risk factor for fracture [9, 10], may contribute to the increased fracture rates found in CD [11]. The temporal relationship of fracture to diagnosis of CD has however not been widely studied. One report of female patients over 50 years suggested that an excess of nonspine, nonwrist fractures occurred in the period from 10 years before until 5 years after diagnosis [12]. Given the frequent delay in diagnosis of CD until recent times and the low incidence of hyperparathyroidism, the possibility arises that nonparathyroid related bone fragility is present for some time before diagnosis. Two studies of lower tibial volumetric BMD may provide further reason to suspect that hyperparathyroidism is not the only factor affecting bone density in coeliac disease. These studies of trabecular and cortical bone in the peripheral skeleton in newly diagnosed patients with CD have disclosed a dichotomy in the response of trabecular and cortical bone density at the distal tibia using pQCT [7, 13]. Both reported a reduction in trabecular density, but only one study found a deficit of cortical density [13]. Patients in the study with lower cortical thickness had lower values for body weight, serum calcium, and 25(OH)D and higher values for PTH and for the bone collagen breakdown marker cross-linked C-telopeptide (CTx) [13] compared with the study in which preservation of cortical thickness was associated with normal values for PTH, 25(OH)D, and CTx [7]. Thus although secondary hyperparathyroidism may play a role, it may be less relevant for trabecular bone at the distal tibial site.

A further difference between the two pQCT studies lay in the patients' body weight. Although neither study showed a difference between body weight in control or CD subjects, the study reporting a low cortical thickness included patients whose body weight was almost 7 kg lighter [13] than in the other study [7]. In normal subjects low areal BMD is associated with low body weight [14]. In a previous study of CD, only nonwrist fractures had been in excess in the period from 10 years before diagnosis until 5 years after diagnosis [12].

In the present study we investigated areal BMD (BMD) in 6 regions of the lower limb to determine whether deficits of bone in the epiphyseal/metaphyseal region (largely trabecular) or diaphyseal regions (largely cortical bone) occurred in the lower limbs of newly diagnosed patients with CD and to establish whether any deficit was associated with increased bone turnover, hyperparathyroidism, or body weight. Since oestradiol status may affect parathyroid hormone action in primary hyperparathyroidism [15, 16] and PTH values in secondary hyperparathyroidism [17] studies were confined to men who are a relatively understudied group in CD.

## 2. Patients and Methods

### 2.1. Patients

Twenty-eight male patients aged 38–77 yr with CD agreed to the analysis of their lower limb bone mineral density (LL-BMD) from the digitally stored whole body dual energy X-ray absorptiometry (DXA) scan. This pilot study exploited additional retrospective analysis on DXA scans already acquired for routine management. All patients were within 6 months of diagnosis of CD and had been referred for routine metabolic bone assessment at a specialist orthopaedic hospital. Coeliac disease in the patients either had been diagnosed by the referring gastroenterologist or was diagnosed at referral by serological testing. No details of intestinal biopsy, Marsh grading, or genetic assessment were available. Patients were asked about use of calcium supplements, fractures, and conditions that might affect bone health and BMD. Control subjects were screened by health questionnaire to exclude diseases or medications known to affect bone metabolism but subjects with a history of fracture were accepted and were taken from a larger study of osteoporosis in men [18, 19]. All patients and control subjects were Caucasian and were resident within the referral area of the hospital.

### 2.2. DXA Measurements

BMD was measured by Hologic 4500A (Hologic Inc. Waltham, MA, USA) at 2nd lumbar to 4th lumbar vertebra (L2–4) inclusive and at the femoral neck (FN) according to established protocols with daily quality control. Whole body DXA scans were obtained according to the manufacturer's protocol by experienced DXA practitioners who ensured precise and consistent positioning of patients and subjects on the scanning table. Weight was taken from the whole body composition DXA reading (grams) and converted to kilograms and height was measured to the nearest 0.1 cm using a Harpenden Stadiometer (Holtain, Crymych, UK). Body mass index was measured as kg/m^2^.

**Figure 1:**
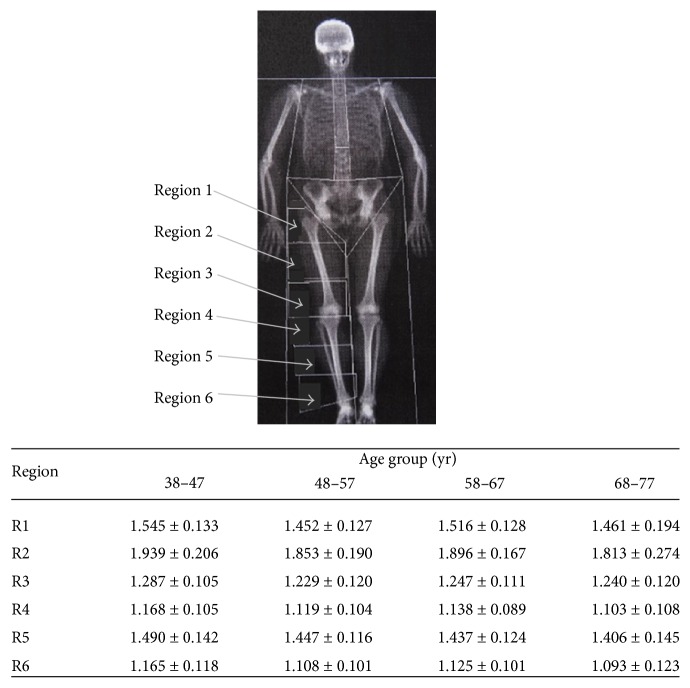
Designation of regions of the lower limb skeleton (Regions 1–6) with data for control subjects. Only the regions on the right side are depicted in the figure. The associated table shows the mean bone mineral density measurements for the control group from which *z* scores were calculated. The measurements show the average of the right and left side combined (mean ± SD) according to region and age group.

Lower limb analysis was undertaken from the whole body DXA scan [20]. In brief each lower limb was divided into six regions from a site through the neck of the femur to the inferior end of the tibia/fibula and the (areal) BMD calculated for each region.  Figure 1 depicts the regions of the lower limb analysed together with areal BMD values for the healthy controls in each of the regions. Regions 2 and 5 are largely diaphyseal and cortical bone, whereas Regions 3, 4, and 6 represent metaphyseal bone with a greater proportion of trabecular bone. Average BMD values for each lower limb region were calculated by dividing the combined values of bone mineral content for right and left side for each region by the combined areas. Data from control subjects over the same age range (see above) were used to establish the normal ranges for spine, femoral neck, and lower limb regions.

For BMD analyses, data in patients with coeliac disease were converted into *z* scores {(patient BMD − age matched control BMD)/age matched standard deviation}. with patients and controls matched in 10-year age groupings aged between 38 and 77 yr. For these calculations the control subjects were divided into four ten-year groupings (38–47 yr *n* = 33; 48–57 yr *n* = 43; 58–67 yr *n* = 40; 68–77 yr *n* = 17) or for comparative purposes at the femoral neck values were also taken from the relevant age groups in Looker et al. [21].

In addition for some comparisons, BMD was also derived not only from age but also by weight. Patients and control subjects in each age group were divided into above and below average weight and a *z* score (denoted BMD *z*_wc_) based on both age and body weight was calculated from those control subjects who were above or below average body weight for the coeliac cohort. In addition *T* scores were calculated using values from the control group. The highest BMD in Regions 1–6 occurred in the 30–39 yr age group, whereas for the lumbar spine and femoral neck the highest values were found in the 20–29 yr age group. Each age group was divided into those above or below average weight and a weight corrected *T* score was applied with values derived from the control subjects divided by weight as in the derivation of weight corrected *z* scores. In addition for comparative purposes femoral neck *T* scores were also calculated from the data of Looker et al. [21] although these are not weight corrected.

### 2.3. Biochemical Measurements

Serum calcium (Ca), thyroid stimulating hormone (TSH), albumin, serum total alkaline phosphatase (ALP), and urinary Ca and creatinine (Cr) (measured from 24 hr urine collections with patients and controls taking their usual diet) were determined using standard methods by the Shropshire Hospitals Pathology Service. The range for serum ALP in normal subjects was established by taking the logarithm of the ALP value and calculating the mean and standard deviation from these data and using the antilog data for the mean and standard deviation. Serum TSH was only undertaken in the coeliac subjects, the range in healthy normal subjects being 0.5 to 4.5 IU/L. Serum intact PTH was measured from EDTA samples using a Siemens Immulite 2000 analyser (Siemens Medical, Los Angeles, Ca, USA). Mean and standard deviation values for PTH were calculated similarly to serum ALP values and log⁡PTH was used in correlations. Blood samples were obtained in both CD patients and controls between 9.30 am and 4.00 pm during routine and research clinic hours. Urinary N-telopeptide (NTx Osteomark, Ostex International Inc., Seattle, WA, USA) and creatinine were measured as previously described [22] on 2nd morning urine samples and the results expressed as nmol bone collagen equivalents (BCE)/mmol creatinine. Values for vitamin D metabolites were not available.

### 2.4. Statistics

Since the number of patients required for this study was unknown, an assumption was made that the difference in BMD in the lower limb regions would be similar to that found between control subjects and coeliac patients at the femoral neck and that the standard deviations would be similar. The femoral neck was selected as being that recommended for the definition of osteoporosis [23]. The difference between the means at the femoral neck of coeliac and controls was found to be 8% and this percentage reduction was applied to each region in the lower limb. The difference between the mean value for each region and a value 8% lower was then used to calculate the number of patients required for significance at the 5% level with a power of 80% at each region [24]. These calculations suggested that the number of patients required to achieve significance was between 18 and 26 depending on the region.

Normality of a data series was examined by the Schapiro-Wilk test. Normally distributed data were compared using Student's *t*-test for two independent samples when two variables were being compared. In the lower limb the null hypothesis was that the values of *z* scores should not differ significantly from zero: hence a one-sample Student's *t*-test was used when *z* scores were being compared with zero. However when *z* scores between two different groups or at two different sites were being compared, Student's *t*-test for two independent samples was used. In addition an effect size was calculated for the differences between the means of the BMD in the control and the coeliac groups for each region examined using Cohen's *d*. A value >0.5 indicates a moderate effect and a value over 0.8 a large effect. Nonnormally distributed data were investigated by the Mann–Whitney *U* test. For associations either the correlation coefficient or the Spearman ranking procedure was followed according to the normality of the data. Data are reported as mean ± standard deviation (SD) and as median (interquartile range) for nonparametric data (apart from serum ALP and PTH,* see above*). Given the number of regions involved, the number of comparisons being made, and the number of patients in the coeliac group, a Bonferroni correction without any modification was applied. Proportions were compared using the Fisher exact test. Significance was assumed at the 5% level.

### 2.5. Ethics

Ethical permission for the studies on the coeliac patients was obtained from the NRES committee, Hampshire B site, UK, on 14 October 2014 under the study title “Analysis of the Lower Limb in Historic Bone Density Scans for Bone, Lean and Fat in Patients with Coeliac Disease” (REC reference 14/SC/1199, IRAS Project number 161019). Each patient had an information sheet and informed consent was obtained from each patient according to the ethics committee requirements. Data on control subjects [18, 19] had been collected in the course of other (published) studies for osteoporosis under permission from the North Wales (East) and North Staffordshire ethics committees.

## 3. Results

Patients with coeliac disease (*n* = 28) had a number of associated conditions including anaemia (*n* = 5), Addison's disease (*n* = 1), a previous history of thyroid disease (*n* = 2), diabetes mellitus (*n* = 2), ECG changes (1 with atrial fibrillation and 1 with long QTc syndrome), and neurological or psychiatric abnormalities (*n* = 2). Two patients had been found on screening because of a family history. Four patients took 21 units or more of alcohol weekly and 10 had sustained fractures. Five coeliac patients were taking calcium supplements.

Baseline data for the coeliac and control subjects are presented in Table 1. There was no significant difference in age, weight, or height between the two groups. BMI (range 19.9–31.8 kg/m^2^) and weight (range 63.7–98.3 kg) were slightly lower in coeliac patients without achieving significance. In the biochemical measurements coeliac patients had higher ALP than controls (although only 3 values were more than 2 standard deviations above the range for normal subjects), but values for serum calcium, albumin, urinary Ca/Cr, and NTx/Cr were all similar to controls. No significant associations between NTx/Cr or ALP and BMD *z* scores in the lower limb regions in coeliac patients (Spearman rho testing) were found. Urinary Ca excretion/day showed no relationship with body weight or with BMD *z* score at any lower limb region.

Three patients had PTH values more than 2 SD above the control mean (>8.85 pmol/L), but overall, values were not significantly different from controls. Log (urinary NTx/Cr) was positively associated with log⁡(PTH) values (*r* = 0.47; *p* < 0.02). Otherwise no relationship was found between either BMD *z* score at any lower limb region or body weight or urinary Ca/Cr and log PTH values.

In the lower limb regions, control subjects showed a slight decline in BMD with age and were divided into four 10-year age groups from 38 to 77 yr to give reference data for use in calculating *z* scores. In the coeliac patients, BMD *z* scores in Regions 1, 4, and 6 were low after Bonferroni correction (Table 2) and the magnitude of the difference was reflected in the effect size. Compared with Region 2, Region 6 (*p* = 0.014) and Region 4 (*p* = 0.038) had lower BMD *z* scores, and BMD *z* score in Region 6 was lower than in Region 5 (*p* = 0.038) (two-sample *t*-test).

BMD *z* score at the femoral neck in coeliac patients was lower than in the controls, but that at the spine showed no significant difference. BMD Region 6 *z* score was significantly lower than either the lumbar spine *z* score (*p* = 0.014) or the femoral neck *z* score (*p* = 0.026; two-sample *t*-test).

**Figure 2:**
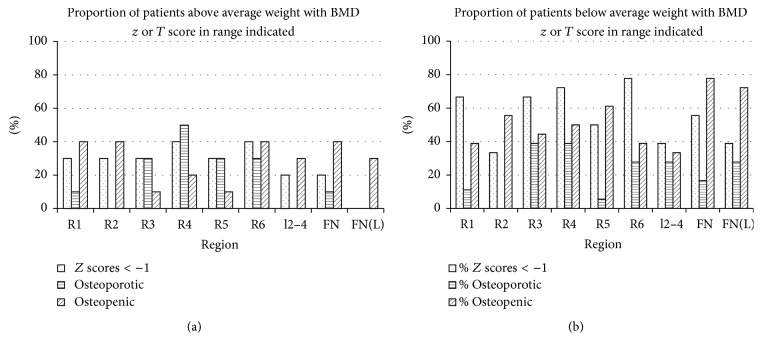
Proportion (as %) of patients with osteoporosis (*T* score −2.5 or lower), osteopenia (*T* score −1 to −2.499), or a *z* score below −1. R1–R6 indicate the regions of the lower limb (see Section 2). l2–4 refers to lumbar vertebrae 2–4 inclusive; FN: femoral neck. FN(L) refers to *T* scores calculated from the data of Looker et al. 1997. *Z* and *T* scores were calculated as described in Section 2 from control subjects [18, 19] or for FN(L) from [21]. (a) Patients with above average weight and (b) patients with below average weight.

In the control group, BMD was positively associated with body weight in each lower limb region and for each age group. To assess whether low body weight in CD patients might be associated with low BMD *z* scores, patients were divided into those above average weight (*n* = 10, age 58 ± 9 yr) and those who were below average weight (*n* = 18, age 61 ± 13 yr; *p* = ns) of the controls (see Table 1). As BMD in control subjects was related to weight in all lower limb regions, *z* scores were then recalculated in the controls not only according to age but also according to whether controls were above or below average weight in each age group (see Section 2). This gave a *z* score corrected for above or below average body weight (weight corrected *z* score = BMD *z*_wc_). The BMD *z*_wc_ scores (Table 3) show that only the CD patients with low body weight exhibited low BMD *z*_wc_ scores. Figures 2(a) and 2(b) show the proportion of patients with low BMD *z* scores, osteoporosis (*T* ≤ −2.5), and osteopenia (*T* score −1 to 2.499).  Figure 2(a) depicts values in coeliac patients above average weight and Figure 2(b) those patients below average weight. The greater effect of low body weight on the prevalence of low *z* and *T* scores is evident even with values in the control group being weight corrected (see Section 2).

The slopes and intercepts of the lower limb BMD *z* scores versus weight were similar to those in the spine and the femoral neck. Region 2 (largely cortical bone) and Region 6 (with a greater proportion of trabecular bone) correlated with body weight (Region 2 *z* score = 0.06 × body weight + 5.1; *r* = 0.45  *p* < 0.02); (Region 6 *z* score = 0.065 × body weight + 6.3; *r* = 0.45, *p* < 0.02). These relationships were similar to the lumbar spine (*z* score = 0.076 × body weight + 6.4; *r* = 0.6) and the femoral neck (*z* score = 0.078 × body weight + 6.6; *r* = 0.6).

Data for patients were also examined according to the presence or absence of a history of fracture (at any site). Patients who had fractured were of similar weight compared with those without fracture, and generally had lower LL-BMD *z* scores for all regions, but only at Regions 4 and 5 were values significantly lower (Region 4 *p* = 0.044; Region 5 *p* = 0.045) but neither survived a Bonferroni correction. Four patients had sustained an ankle fracture, but in this small sample there was no difference in BMD between the two lower limbs at Region 6 (lower leg). More patients with PTH above the mean (6 out of 14) had a history of fracture compared with patients whose PTH fell below mean values (2 out of 12) but the difference (Fisher exact test) was not statistically significant.

## 4. Discussion

This group of older male patients with CD showed a reduction of areal BMD at the distal lower leg (Region 6), a site similar to that recorded recently by pQCT in studies of younger females with CD [7, 13]. The present study also extended the sites of investigation by including those with predominantly cortical bone. These sites were less affected by the coeliac process than the mainly trabecular sites. Region 3, although showing a significant deficit of bone in the coeliac patients, did not survive a Bonferroni correction. This may arise through the incorporation of a greater proportion of cortical bone at this site.

Overall however regions with a greater proportion of trabecular bone had lower BMD *z* scores compared with regions having a predominance of cortical bone. Whilst the early involvement of trabecular bone may reflect the more rapid turnover in trabecular bone, it may also indicate earlier involvement of trabecular bone in the coeliac process. Further it may be independent of significant osteoclastic activity as evidenced by the normal values for bone resorption markers in this study and that of Stein et al. [7]. Moreover we were unable to find relationships with PTH for either cortical or trabecular BMD *z* scores in the lower limb in contrast to the findings of Selby et al. in a similar number of patients, albeit largely female, at the distal forearm [2]. The data of Selby et al. [2] differ from ours in the fact that their patients had been diagnosed almost 10 years previously, whereas the present patients were within 1 year of diagnosis. A further report of treated coeliac patients also pointed out that the correlation between BMD and PTH depended on a very few high values for PTH [25]. The mean value for PTH in our patients was not elevated, as in the data of Stein et al. [7]. Other reports have expressed high PTH in various ways but our finding of 3 out of 24 (12.5%) above mean + 2 SD or 6 out of 24 (25%) with values above 7.0 pmol/L are similar to previous reports [5, 6].

More significant however was the relationship of low BMD to low body weight in all regions, although Region 6 failed to withstand a Bonferroni correction (Table 3) suggesting that additional factors might be important in the lower leg. The similarity of the relationships between the spine, femoral neck, and Regions 2 and 6 and body weight suggests that coeliac patients follow a similar relationship between bone and body weight as has been described in normal subjects [14], but the normal values for serum albumin indicate that malnutrition is unlikely. We were not able to discern whether our patients had lost weight or never gained weight. It is possible that those patients with the lowest body weights had the greatest degree of malabsorption, but if so the weight deficit was not related either to elevated PTH values or to low UCa (it is not appropriate to relate body weight to urinary Ca/Cr since weight and urinary creatinine are correlated). The biochemical data all suggest that bone turnover was not increased in our male subjects concurring with data for male and female subjects in earlier reports [5, 6].

Low body weight however may only be one factor contributing to low BMD in CD. Anti-osteoprotegerin antibodies have been found in CD (leading to higher values for RANKL and hence greater bone breakdown) [26] whilst a study from Malta suggested an inflammatory association of low BMD at the hip and spine since adequate sunlight exposure made vitamin D deficiency unlikely even though malabsorption was present [27]. Patients with CD have low appendicular nonbone lean mass [28], a feature in common with patients affected by inflammatory bowel disease in whom low appendicular muscle mass is associated with osteopenia [29]. In Crohn's disease muscle wasting was reversed by the use of Infliximab [30], suggesting an inflammatory element to the muscle wasting. More recently alteration in the gut microbiome has been found to affect BMD in animal models [31]. Thus more than one process may be involved in the loss of bone in the lower limb in CD, only one of which may be hyperparathyroidism. This process particularly appears to affect the more trabecular sites in the lower limb and is associated with low body weight. In our patients, values for the cross-linked bone collagen breakdown marker NTx/Cr were not elevated. This may indicate the slow rate at which bone was being lost. A history of previous ankle fracture in our patients did not appear to affect measurements in Region 6. There was a trend for patients with a history of a preexisting fracture at any site either to have a lower BMD *z* score at lower limb regions or to have above median values for PTH, but with the present number of patients neither association was significant.

The present study has several limitations. It was conducted in a clinical setting and in a metabolic bone disease department which provided a service to other specialties for metabolic and endocrine problems. The numbers in this pilot study were limited and further research may clarify whether weight is important in Region 6. Studies with a greater number of patients would clarify the relative contributions of high PTH values or low BMD values in the lower limb to the prevalence of ankle or other peripheral nonwrist fractures. It was not possible to obtain details of Marsh grading or genetic testing. In addition a number of measurements that would have been useful were outside the funding for the study. Titres for anti-tTG were not available in many patients, although the relationship of anti-tTG titre and bone involvement is doubtful [7, 32]. Bone specific ALP was not measured and thus the source of the slightly high serum ALP could not be determined. 25(OH)D was not measured because some studies had shown no correlation with BMD [2]. We did not measure testosterone, a hormone that is anabolic to bone, possibly through conversion to estradiol [33]. Resistance to testosterone has been described in coeliac disease [34] and if resistance exists downstream to estradiol, low BMD in female patients might also be expected. DXA analysis of lower limb regions has been more commonly used in patients with spinal cord injury in whom quite large deficits of bone have been described around the knee [35, 36].

In conclusion, these data suggest that factors in addition to hyperparathyroidism may play a role in the deficit of BMD in coeliac disease. Further work relating cytokines and bone turnover markers are indicated together with BMD measurements at regions other than the spine and femoral neck, particularly since, compared with these sites, the distal tibia appears to have a greater deficit of bone. With a greater number of patients, relationship of peripheral fractures to hormonal changes, cytokine levels, and BMD values at nonclassical sites could be pursued. Moreover this can be done using DXA, a technique now widely available, since even with a conservative statistical approach to avoid false positive results, significant results were obtained.

## Figures and Tables

**Table 1:** Baseline characteristics of the male coeliac patients and age matched healthy control subjects.

	Control		Coeliac		*p*
	*N*		*N*		
Age (yr)	133	56 ± 10	28	60 ± 12	ns
Weight (kg)	133	82 ± 11	28	78 ± 9	ns
Height (cm)	133	175.5 ± 6.3	28	175.3 ± 6.5	ns
BMI (kg/m^2^)	133	26.7 ± 3.0	28	25.6 ± 3.0	ns
Serum Ca (mmol/L)	91	2.39 ± 0.08	27	2.37 ± 0.10	ns
Serum albumin (g/L)	131	43.3 ± 2.5	27	42.6 ± 2.7	ns
Serum PTH (pmol/L)	51	*3.5 (2.2–5.6)*	*24*	*4.4 (2.0–8.9)* ^§^	ns^†^
Serum alkaline phosphatase (iu/L)	131	*62 (48–80)*	*26*	*79 (65–96)* ^§^	0.01^†^
UCa/Cr (mmol/mmol)	75	0.32 (0.19–0.43)	26	0.19 (0.12–0.41)^¶^	ns^†^
UNTx/Cr nmol BCE/mmol Cr	81	38.9 (26.9–50.1)	27	36.2 (23.3–46.3)	ns^†^
TSH (mu/L)		Not available	24	1.86 (1.52–2.41)^Ø^	

^*Ø*^Normal range for TSH is 0.5–4.5 mu/L.

^¶^Excluding 5 patients taking oral calcium supplements.

^§^Serum PTH and ALP values are mean ± 1 standard deviation of the antilogged values (see Section 2).

^†^Mann–Whitney *U* test.

Values are mean ± SD or for the italicized data median and range.

**Table 2:** Bone mineral density in the lower limb regions, spine, and femoral neck in patients with coeliac disease compared with controls.

Region	Control BMD (g/cm^2^)^¶^ (*n* = 133)	Coeliac BMD (g/cm^2^) (*n* = 28)	Coeliac *z* score	*p* *z* score^†^	*p* with Bonferroni correction	Effect size Cohen *d*	Number of coeliac patients with negative *z* score
1	1.476 ± 0.133	1.328 ± 0.143	−0.940 ± 0.974	0.0001	0.0006	1.13	23
2	1.891 ± 0.192	1.796 ± 0.252	−0.33 ± 1.214	ns	—	0.45	21
3	1.253 ± 0.113	1.163 ± 0.187	−0.765 ± 1.649	0.02	0.12	0.51	21
4	1.136 ± 0.099	1.018 ± 0.152	−1.093 ± 1.448	0.0004	0.0024	0.95	22
5	1.457 ± 0.128	1.376 ± 0.195	−0.418 ± 1.491	0.1134	—	0.5	21
6	1.130 ± 0.105	0.982 ± 0.147	−1.190 ± 1.292	0.0001	0.0006	1.22	23
Lumbar spine	1.065 ± 0.154	1.010 ± 0.167	−0.372 ± 1.110	ns	na	0.35	19
Femoral neck	0.847 ± 0.127	0.778 ± 0.142	−0.456 ± 1.099	0.038	na	0.51	20

^¶^Control BMD refers to average across whole age range 38–77 yr. ^†^Probability of coeliac *z* score being less than zero (one-sample *t*-test). ns: not significant. na: not applied. Control data are from [18, 19]. Effect size is an indication of how great the difference is between 2 measurements and is independent of sample size. Above 0.8 indicates a large difference, above 0.5 a medium difference, and below 0.5 a small difference.

**Table 3:** BMD *z*_wc_ scores for coeliac patients above and below average weight.

Region	Below average weight (*n* = 18)		*p* before Bonferroni ×6	*p* after Bonferroni ×6^†^	Above average weight (*n* = 10)		*p* before Bonferroni ×6	*p* after Bonferroni ×6^†^
	Mean	SD			Mean	SD		
1	−1.259	1.032	0.0001	0.0006	−0.806	1.194	0.06	ns
2	−0.614	1.223	0.048	ns	0.205	1.484	0.67	ns
3	−1.330	1.338	0.0006	0.0036	0.351	1.878	0.56	ns
4	−1.931	1.188	0.0001	0.0006	−0.427	1.193	0.5	ns
5	−0.893	1.002	0.0015	0.009	0.2	1.799	0.73	ns
6	−1.187	1.327	0.0014	0.008	−1.077	1.511	0.051	ns

BMD *z*_wc_ refers to the BMD corrected both for age and for above or below average weight in the controls (see text).

† refers to probability value after Bonferroni correction for six observations.

ns: not significant.
